# Productivity and carcass characteristics of lambs fed fibrous agricultural wastes to substitute grass

**DOI:** 10.14202/vetworld.2021.1559-1563

**Published:** 2021-06-17

**Authors:** Endang Purbowati, C. M. Sri Lestari, Retno Adiwinarti, Vita Restitrisnani, Sri Mawati, Agung Purnomoadi, Edy Rianto

**Affiliations:** Department of Animal Sciences, Faculty of Animal and Agricultural Sciences, Diponegoro University, Campus Drh. Soejono Koesoemowardojo, Tembalang, Semarang 50275, Indonesia

**Keywords:** bagasse, corn cobs, digestibility, feed efficiency, meat-bone ratio, peanut shells

## Abstract

**Background and Aim:**

Grass is often scarce for ruminants during the dry season in Indonesia; thus agricultural by-products are widely used as a substitute for grass. This study aimed to determine the effect of replacing Napier grass (NG) with agricultural by-products on the productivity and carcass characteristics of lambs.

**Materials and Methods:**

Twenty-four 3-month-old male lambs with initial body weights of 13.26±1.29 kg (coefficient of variation=9.73%) were allocated into a completely randomized design with four treatments and six replications. The treatments included: NG=100% NG; corn cobs (CCs)=50% NG and 50% CCs; bagasse (BG)=50% NG and 50% BG; and peanut shells (PSs)=50% NG and 50% PSs. All treatment diets were pelleted and consisted of 40% fibrous feed and 60% concentrate feed, and contained 10.36-11.65% crude protein and 55.47-57.31% total digestible nutrients. Parameters observed included dry matter intake (DMI), dry matter digestibility, body weight gain (BWG), feed conversion ratio (FCR), feed cost per gain (FC/G), and carcass characteristics.

**Results:**

Lambs fed the PSs diet had the highest (p<0.05) DMI (781 g/d), digestibility, and body weight gain (92.5 g/d; p<0.05). The FCR of the PSs diet (9.13) was similar to NG. The FC/G of the PSs diet (IDR 23,541/kg) was the lowest of all diets. The BG diet had the lowest (p<0.05) digestibility, body weight gain (54.4 g/d), and the highest (13.53) FCR. No significant differences (p>0.05) were found in the carcass or meat characteristics of any diets. The averages of slaughter weight, carcass weight, and carcass percentage were 20.03 kg, 8.02 kg, and 40.0%, respectively. The average meat bone ratio was 3.67.

**Conclusion:**

It was concluded that agricultural wastes could be used as an alternative to NG at the level of 50% in the diet of lambs without a negative effect on production performance and carcass traits.

## Introduction

Agricultural by-products are widely used for ruminant feed when grass is limited during the dry season in some countries. The use of agricultural waste as a source of fiber for ruminants can reduce feed prices and improve environmental sustainability using feed materials that are sustainably available. Examples of agricultural waste include corn cobs (CCs) [1-3], bagasse (BG) [4-7], and peanut shells (PSs) [8]. Ground CCs have been used as a fiber source in cows [1], goats [2], and lambs [3]. Babiker *et al*. [5] used BG for beef cattle, while Silva *et al*. [4], Filho *et al*. [6], and Galvani *et al*. [7] used it for lambs. Eshag *et al*. [8] used ground PSs for lambs to reduce feed cost.

Agricultural by-products are usually categorized as low-quality roughages due to their high crude fiber, neutral detergent fiber (NDF), and acid detergent fiber (ADF) contents. CCs contain 10.2% crude fiber, 38.0-79.9% NDF, and 16-40% ADF [2,3]. BG contains 84.6% NDF and 53.1% ADF [7]. PSs contain 43.9%-80.5% crude fiber, 27.6-87% NDF, 13.1-76.2% ADF, and 5.8-45.2% lignin [9]. These by-products have been processed into many dietary forms, such as ammoniated BG, BG treated with calcium oxide [6], ground maize cobs [1], and maize cob silage [3]. Wachirapakom *et al*. [1] used ground CCs as a single roughage source and found that ground CCs result in better dry matter intake (DMI) and milk yields compared to rice straw when used as a source of fiber in dairy cow diets. Treated agricultural by-products have additional positive effects on ruminants when used as a roughage source, including decreased methane emission in lambs [3] and improved nutrient intake and milk yield in lactating dairy, crossbred cows [1]. Complete feed or total mixed ration (TMR) is the ideal method to blend concentrate and roughage from local agricultural by-products to make a cheap and balanced ration [10]. Blended feed material in TMR can improve feed palatability [10-12], which allows unconventional feeds to be added that further reduce the price of the ration [10]. The forage and concentrate ratio in TMR can be formulated to meet the animals’ nutritional requirements for the desired health status and production performance [12]. For example, Lin [12] found that equal amounts of proteins and carbohydrates in TMR maximize the fermentation activity of ruminal microbes and stabilize rumen pH. The further processing of TMR into pellets increases feed intake and digestion [10]. Karimizadeh *et al*. [13] reported that the DMI of lambs fed pelleted TMR was higher than those fed mash TMR (1.2 g/d vs. 1.1 g/d, respectively). Islam *et al*. [14] also reported that pelleted TMR increased crude fiber digestibility compared to mash TMR. Ishaq *et al*. [15] found that lambs fed pelleted diets had an increased DMI of around 17% compared to those fed loose hay diets (1.9 kg/d vs. 1.6 kg/d, respectively), with a higher average daily gain (ADG; 0.24 kg/d vs. 0.08 g/d, respectively).

This study investigated the use of agricultural by-products as a component of pelleted lamb feed to increase productivity and reduce feed costs (FC), since agricultural by-products are abundantly available to substitute grass and may be best utilized as pellets.

## Materials and Methods

### Ethical approval

The animal handling and scientific procedures in this study were approved by the Animal Ethics Committee from the Faculty of Animal and Agricultural Science, Diponegoro University, Indonesia.

### Study period and location

The study was conducted from July to December 2019 at the Research Farm of Meat and Dairy Production Laboratory, Department of Animal Science, Faculty of Animal and Agricultural Sciences, Diponegoro University, Semarang, Indonesia.

### Animals, experimental design, and treatments

The study used 24, 3-month-old male lambs with an average body weight of 13.26±1.29 kg (coefficient of variation=9.73%). The lambs were allocated into a completely randomized design [16] with four treatments and six replications. The diets contained three different agricultural by-products (i.e., CCs, BG, and PSs) as a fiber source to substitute Napier grass (NG). The concentrate consisted of rice bran, cassava waste product, soybean meal, molasses, and minerals. The diets consisted of 40% fibrous feedstuffs and 60% concentrate, and were formulated to contain 10.36-11.65% crude protein and 55.47-57.31% total digestible nutrients (TDN) (Table-1) [17]. The diets were offered to the animals in pellet form. The treatments included NG=100% NG (with no agricultural wastes); CCs=50% NG and 50% CCs; BG=50% NG and 50% BG; and PSs=50% NG and 50% PSs.

**Table-1:** Feed composition and nutritional content of the diets.

Feed composition/nutritional content	NG	CCs	BG	PSs
Feed composition (%)				
Napier grass	40.00	20.00	20.00	20.00
Corn cobs	0.00	20.00	0.00	0.00
Bagasse	0.00	0.00	20.00	0.00
Peanut shell	0.00	0.00	0.00	20.00
Rice bran	34.05	34.05	34.05	34.05
Cassava waste products	5.00	5.00	5.00	5.00
Soybean meal	12.95	12.95	12.95	12.95
Molasses	6.00	6.00	6.00	6.00
Mineral mix	2.00	2.00	2.00	2.00
Nutritional content (%)				
Dry matter	92.20	92.42	92.14	92.17
Ash	9.75	8.85	8.46	9.96
OM	90.25	91.15	91.54	90.04
CP	11.65	10.73	10.36	10.41
EE	5.55	6.08	5.19	5.23
CF	31.43	33.16	34.81	37.46
NFE	41.62	41.18	41.18	36.94
TDN	56.48	57.31	55.47	55.77
NDF	55.83	57.61	54.47	56.76
ADF	27.38	29.63	31.20	32.15
Price (IDR/kg)	2,352	2,352	2,331	2,377

NG=100% Napier grass, CCs=50% Napier grass and 50% corn cobs; BG=50% Napier grass and 50% bagasse, PSs=50% Napier grass and 50% peanut shells, TDN was calculated from the formula TDN=digested CP+(2.25×digested EE)+digested CF+digested NFE [17]. OM=Organic matter, CP=Crude protein, EE=Ether extract, CF=Crude fiber, NFE=Nitrogen-free extract, TDN=Total digestible nutrients, NDF=Neutral detergent fiber, ADF=Acid detergent fiber

### Observed variables, research activities, and procedures

The examined variables included lamb productivity and carcass characteristics (i.e., DMI, organic matter intake (OMI), dry matter digestibility (DMD), organic matter digestibility (OMD), body weight gain (BWG), feed conversion ratio (FCR), FC per gain (FC/G), carcass traits, carcass composition (bones, meat, and fat), and meat-bone ratio). The experimental period consisted of five stages: Preparation (3 weeks), adaptation (3 weeks), the preliminary period (1 week), the treatment period (12 weeks), and slaughtering. The lambs were provided the diets and water *ad libitum* and were weighed once a week. Feces were collected for 7 days during week 3 of the treatment period to calculate feed digestibility [18]. The feces were collected daily, weighed, and sampled using the procedure of Darlis *et al*. [19]. After the 12-week treatment period, the lambs were fasted for 12 h with free access to freshwater, and then slaughtered. The carcasses were weighed and separated for bone, fat, and meat according to Pratiwi *et al*. [20]; however, in this study, the kidneys were included in the carcasses.

### Statistical analysis

The data obtained were analyzed using analysis of variance, apart from the FC/G, which was analyzed descriptively. Duncan’s multiple range test was applied when there were differences among the treatments [16]. The level of significance was based on p<0.05.

## Results

### Lamb’s productivity

The effect of NG substitution by agricultural by-products on lamb productivity is presented in Table-2. The DMI and OMI of the lambs fed the PSs diet were significantly higher (p<0.05) than the other diets, although the DMI in the percentage of body weight was not significantly different (p>0.05) among the treatments. The crude protein intake (CPI) of lambs fed the PSs and NG diets was higher (p<0.05) than that of the CCs- and BG-fed lambs. The CPI of the lambs fed the PSs and NG diets was not significantly different (p>0.05). The crude fiber intake (CFI) of the lambs fed the NG diet was the lowest. The TDN intake of the lambs fed the NG and BG diets was lower than that of those fed the PSs diet. The TDN intake of the lambs fed the CCs diet was not significantly different (p>0.05) from those fed the NG, BG, and PSs diets.

**Table-2:** Productivity of lambs fed diets with different sources of fiber.

Variables	NG	CCs	BG	PSs
Dry matter intake (g/days)	724^a^	722^a^	718^a^	781^b^
Dry matter intake (% BW)	4.42	4.44	4.50	4.73
Organic matter intake (g/days)	654^a^	658^a^	657^a^	704^b^
Crude protein intake(g/days)	84^b^	76^a^	74^a^	81^b^
Crude fiber intake (g/days)	228^a^	240^b^	250^b^	293^c^
TDN intake (g/days)	410^a^	416^ab^	398^a^	435^b^
Dry matter digestibility (%)	51.9^ab^	51.4^a^	50.9^a^	53.0^b^
Digestible dry matter intake (g/days)	376^b^	372^ab^	365^a^	414^c^
Organic matter digestibility (%)	55.9^ab^	53.9^a^	52.8^a^	62.1^b^
Digestible organic matter intake (g/days)	367^a^	355^a^	347^a^	437^b^
Body weight gain (g/days)	77.5^bc^	68.2^ab^	54.4^a^	92.5^c^
Feed conversion ratio	9.7^a^	11.2^ab^	13.5^b^	9.1^a^
Feed cost per gain (IDR/kg)	24,835	28,554	34,234	23,541

^a,b,c^Within a row, means without a common uppercase superscript differ (p<0.05). NG=100% Napier grass, CCs=50% Napier grass and 50% corn cobs; BG=50% Napier grass and 50% bagasse, PSs=50% Napier grass and 50% peanut shells. BW=Body weight

The DMD and OMD of the PSs diet were higher (p<0.05) than those of the CCs and BG diets, but were not significantly different (p>0.05) from those of the NG diet. There was no significant difference (p>0.05) in DMD and OMD for the NG, CCs, and BG diets. The digestible DMI and OMI in the PSs group had the highest value (p<0.05).

The BWG of the lambs fed the PSs diet was higher (p<0.05) than those fed the CCs and BG diets, but was not significantly different (p>0.05) from those fed the NG diet. The lambs fed the NG diet had higher (p<0.05) BWG than those fed the BG diet; however, there was no significant difference (p>0.05) in BWG between the lambs fed the NG and CCs diets, or between the lambs fed the CCs and BG diets (Table-2). Consequently, the FCR was lowest in the PSs-fed lambs and highest in the BG-fed lambs (p<0.05). The BWG and FCR results influenced the FC/G. The lambs fed the PSs diet had the lowest FC/G, followed by those fed the NG, CCs, and BG diets, respectively.

### Lamb carcass characteristics

The effect of NG substitution by agricultural wastes on lamb carcass characteristics is presented in Table-3. No significant differences (p>0.05) were found between the different treatments for any of the measured lamb carcass characteristics. The average slaughter weight was 20.03 kg, which produced 8.02 kg carcass weight and 4.50 kg meat. The average carcass bone weight was 1.74 kg. The meat-bone ratio was 3.67 on average.

**Table-3:** Carcass production and characteristics of lambs fed diets with different sources of fiber.

Variables	NG	CCs	BG	PSs	Average
Slaughter weight (kg)	20.22	19.82	19.26	20.84	20.03
Carcass weight (kg)	8.40	7.88	7.46	8.34	8.02
Dressing percentage (%)	41.5	39.9	38.7	40.1	40.0
Carcass components					
Meat weight (g)	4,79	4,53	4,21	4,45	4,50
Meat percentage (%)	57.1	57.6	56.2	53.3	56.0
Bone weight (g)	1.755	1.663	1.672	1.868	1.740
Bone percentage (%)	21.1	21.2	22.6	22.5	21.9
Fat weight (g)	1.466	1.321	1.253	1.654	1.423
Fat percentage (%)	17.3	16.5	16.9	19.8	17.6
Connective tissue weight (g)	385	367	321	368	360
Connective tissue percentage (%)	4.6	4.7	4.3	4.4	4.5
Distribution of carcass fat					
Subcutaneous fat weight (g)	836	695	723	979	808
Subcutaneous fat percentage (%)	57.1	50.5	57.5	59.2	56.1
Intermuscular fat weight (g)	487	485	395	463	457
Intermuscular fat percentage (%)	33.0	39.0	31.7	28.5	33.1
Kidney fat weight (g)	91	92	90	127	100
Kidney fat percentage (%)	6.5	6.7	7.1	7.4	6.9
Pelvic fat weight (g)	51	49	46	84	58
Pelvic fat percentage (%)	3.5	3.8	3.6	4.9	3.9
Meat-bone ratio			
Meat-bone ratio	3.86	3.78	3.47	3.59	3.67
Lean meat-bone ratio	3.00	2.98	2.72	2.65	2.84

NG=Napier grass, CCs=50% Napier grass and 50% corn cobs, BG=50% Napier grass and 50% bagasse, PSs=50% Napier grass and 50% peanut shells

## Discussion

### Feed intake, digestibility, BWG, FCR, and economic implications

The feed intake and digestibility influenced lamb productivity. The PSs-fed lambs had the highest DMI, OMI, and TDN intake values. These findings could be attributed to the high DMD and OMD of the PSs diet. The high DMI and DMD of the PSs diet led to high DDMI, which, in turn, caused the lambs fed the PSs diet to have the highest BWG and lowest FCR, with the cheapest FC/G. The lambs fed the diet containing BG had the lowest BWG. The BWG of the lambs fed the NG diet was similar to those fed the CCs and PSs diets. These results agree with Santos *et al*. [21] who found that BWG was affected by DMI.

The high NDF and ADF contents (Table-1) in the agricultural by-product diets did not significantly decrease lamb productivity. Usually, the higher NDF and ADF contents cause lower feed intake [21]. We speculate that the PSs and CCs were more palatable in this study, since the inclusion of these feedstuffs did not reduce DMI in the lambs. Another explanation may be that the ingredients were all ground, then offered to the animal in pelleted form. Khan *et al*. [22] stated that grinding and pelleting feeds break down cell walls, reduce particle sizes, increase feed density, increase rumen passage rates, and increase DMI. The inclusion of concentrate in the diet improved the nutrient digestibility of the low-quality crop residues, increased growth rate and meat production [23,24], and reduced FC/G compared to conventional feeding systems [24]. Karimizadeh *et al*. [13] reported that the digestibility of pelleted complete feed was higher than that of mash complete feed.

### Carcass characteristics

The substitution of NG by agricultural wastes did not affect slaughter weight, carcass production, meat production, meat-bone ratio, or subcutaneous fat thickness in lambs. These findings were attributed to the fact that the slaughter weights of the lambs fed with different diets were similar.

The above results indicate that CCs, BG, and PSs could be used to substitute NG without a negative effect on production. Similar slaughter weights were associated with similar carcass weights and dressing percentages, as already pointed out by Sabbioni *et al*. [25]. The carcass weight was affected by the slaughter weight. The carcass weights obtained in this study were similar to those reported by Setyaningrum *et al*. [26], where Indonesian thin-tailed sheep weighing 17.5-18.8 kg produced 7.7-8.5 kg carcasses. Other studies reported that lamb carcass weights varied from 15.3 kg to 26.1 kg when originating from a slaughter weight of 34.3 kg-54.1 kg [21,27-30]. Forwood *et al*. [30] reported that heavier live weights produced higher carcass weights and dressing percentages. Dressing percentages in this study (40.03%) were similar to those reported by Setyaningrum *et al*. [26] in thin-tailed sheep. The previous studies reported that the dressing percentages of different lamb breeds varied from 39% to 51% [21,25,26,29,30]. Valizadeh *et al*. [31] showed that the heavier the body weight of the lamb, the higher its carcass percentage.

In general, consumers prefer meat with less fat [32]. Carcass fat percentage in this study (17.61%) was similar to those reported by Santos *et al*. [21] (i.e., 15-18%) and Obeidat *et al*. [27] (i.e., 16.6-20.1%). Lambs with lighter body weight than mutton contain less carcass fat trimming, as reported by Sabbioni *et al*. [25] who found that lamb fat trimmings were 13.04-17.31%, whereas those in mutton were 15.96-19.06%. Thus, consumers believe that lighter lambs produce healthier meat [32]. In fact, Ekiz *et al*. [33] reported that the lean fat ratios of low, medium, and high weight groups (26 kg, 30 kg, and 35 kg) of lambs were similar (3.93-4.25) with a total fat content of 15.24%-16.81%.

Meat-bone ratio is an important parameter because it is related to the edible portion of the carcass. The meat-bone ratio in this study (3.67) was higher than that in Awassi lambs fed up to 300 g/kg layer litter (2.95-3.10) [27], Cornigliese lambs (2.22-3.09), and mutton (3.25-3.52) [25]. The lean meat-bone ratio in this study (2.84) was lower than that reported by Ekiz *et al*. [33] (3.13-3.48).

## Conclusion

It can be concluded that agricultural wastes, namely, CCs, BG, and PS, can be used to substitute grass as a component of lamb diets at the level of 50% without a negative effect on production performance and carcass traits. The use of PSs to substitute NG increased lamb productivity. Carcass trait characteristics were not affected by agricultural wastes.

## Authors’ Contributions

EP and AP: Designed the study, interpreted the data, and drafted the manuscript. ER, RA, CMSL, and SM: Participated in study design and contributed to the preparation and critical review of this manuscript. RA and VR: Did statistical analysis and drafted the manuscript. All authors read and approved the final manuscript.
